# Editorial: Effects of epilepsy on memory—Therapeutic implications, biomarkers, and comorbidities

**DOI:** 10.3389/fneur.2023.1140941

**Published:** 2023-02-09

**Authors:** Yvonne Höller, Rosa Michaelis, Eugen Trinka, Julia Jacobs

**Affiliations:** ^1^Faculty of Psychology, University of Akureyri, Akureyri, Iceland; ^2^Department of Neurology, University Hospital Knappschaftskrankenhaus Bochum, Ruhr University Bochum, Bochum, Germany; ^3^Department of Neurology, Christian Doppler University Hospital, Centre for Cognitive Neurosciences, EpiCARE, Paracelsus Medical University Salzburg, Salzburg, Austria; ^4^Neuroscience Institute, Christian Doppler University Hospital, Centre for Cognitive Neurosciences, Salzburg, Austria; ^5^Alberta Children's Hospital, University of Calgary, Calgary, AB, Canada

**Keywords:** epilepsy, memory, temporal lobe epilepsy, verbal memory, working memory, high frequency oscillations

Epilepsies are well known to affect memory functions (1, 2), depending on multiple factors, such as type and frequency of seizures (3), the site of the epileptic lesion, the spread of epileptic activity over functional brain regions (4), and possibly processes of inflammation (Li H. et al.).

The best investigated memory disturbance in epilepsy is the common complaint about verbal memory problems among patients with temporal lobe epilepsy [TLE; (5)], especially regarding remote memory and its relation to hippocampal dysfunction (Rastogi et al.). Although reports on memory deficits in epilepsy are numerous, the relationship between memory subdomains and the mediating factors for the negative impact of epilepsy on memory is not clear, yet. For instance, it is less well-known that this deficit is not limited to the semantic domain of memory but interacts with working memory. In this Research Topic, Bolocan et al. demonstrated that patients with drug resistant TLE show impaired working memory, and this deficit contributes to learning deficits and verbal memory problems. Furthermore, the interaction between the number of antiepileptic drugs and the performance in verbal learning and story learning is moderated by working memory (Bolocan et al.).

Disturbance of acquiring verbal memory contents in TLE is also a potential consequence of treating TLE patients *via* surgical resection of the seizure generating tissue. Sala-Padro et al. could demonstrate that there might be additional means to predict the extent of memory decline after surgery. The authors focused on pre-operative resting-state connectivity in functional Magnetic Resonance Imaging (fMRI) in relation to verbal learning decline after anterior temporal lobe resection. They found in both, the pathological and non-pathological anterior connectivity pattern a difference between patients who would experience learning decline and those who remained unaffected. These findings suggest using pre-operative connectivity patterns as a predictive biomarker for surgical effects on verbal memory in TLE. Indeed, network-level approaches become more and more popular in epilepsy research. It was suggested that also cognitive impairment in epilepsy should be tackled beyond seizures, i.e., from a system-level perspective based on network analysis (Khalife et al.). One additional aspect has to be kept in mind when a patient complains about memory deficits after surgical intervention. In addition to objective factors that determine performance, the patient's subjective perception of memory functioning can differ from objective measures, which is an effect that interacts with depressive symptoms and quality of life (Mücke et al.). In general, depressive mood, and its bi-directional relationship with epilepsies (6), hampers cognitive functions and especially memory—depression screenings are of utmost importance in the neuropsychological assessment of patients with epilepsy.

Beyond TLE, also other forms of epilepsy such as juvenile myoclonic epilepsy (JME) show decreased verbal intelligence, verbal fluency and reading speed (Rainer et al.). These deficits, even when tested with emotional words, are however very subtle and might not be evident in assessments of brain activity (Rainer et al.), emphasizing the need for accurate neuropsychological assessment.

Furthermore, epilepsy-related sleep disturbances were shown to negatively affect memory (7), for example in children suffering from benign epilepsy with centrotemporal spikes a higher percentage of spike and slow wave duration during non-REM sleep is related to impaired cognitive functions and abnormal deactivation in the medial frontal cortex and the posterior cingulate cortex (Li Y. et al.). High frequency oscillations (HFOs, 80–500 Hz) represent another important biomarker that is related to memory consolidation during sleep, but in patients with epilepsy they appear to be rather related to pathology and can be linked to areas with poor memory function (Bruder et al.). In general, age should be taken into account when assessing HFOs as they might interact with aging and aging-associated pathology (Windhager et al.). This is a topic that needs to be investigated better in the future, especially regarding the increased vulnerability of patients with Alzheimer's disease to experiencing seizures (Windhager et al.). A third biomarker of sleep-related abnormalities in epilepsy that was investigated in this Research Topic are interictal epileptiform discharges (IED) that are coupled with sleep spindles. Okadome et al. found that these IED-coupled spindles correlated with lower sleep-dependent consolidation of procedural memory, as assessed in a fingertapping task.

It is well known that medication affects cognitive function and especially memory in patients with epilepsy (8, 9). Medication can have a positive effect not only on seizures but also on psychiatric symptoms and on cognition, but effects on memory have yet to be shown for innovative treatments such as probiotics (Wang et al.).

This Research Topic contributed to an increased awareness for memory deficits in epilepsies and that the assessment of epilepsy effects on memory and cognition shall not be limited to the verbal domain. It highlighted electrophysiological biomarkers in a classical sense (e.g., spikes) as well as modern network approaches. We know today that epilepsies can affect various memory sub-domains, such as verbal memory, remote memory, working memory, procedural memory, but also emotional responses that are linked to memory subdomains. Therapeutic implications of medication and surgical interventions should be investigated in future research in order to provide a better understanding for the origin and nature of memory problems in the individual patient.

## Author contributions

YH drafted the editorial and was commented upon and approved by the co-editors/co-authors. All authors contributed to the article and approved the submitted version.
